# Non-nutritive sweeteners, the gut microbiome, and tumor immunity: evidence hierarchy and a sucralose-centered translational model

**DOI:** 10.3389/fnut.2026.1868431

**Published:** 2026-08-18

**Authors:** Jie Li, Deng-Hua Li, Yu-Hao Liu, Yan-Peng Zhang, Qi-Qi Guo, Xiao-Jing Zhu, Yu-Run Wang, Yong-Quan Shi

**Affiliations:** 1 State Key Laboratory of Holistic Integrative Management of Gastrointestinal Cancers and National Clinical Research Center for Digestive Diseases, Department of Gastroenterology & Hepatology, Xijing Hospital, Fourth Military Medical University, Xi’an, Shaanxi, China; 2 Department of Postgraduate, Xi’an Medical University, Xi’an, Shaanxi, China

**Keywords:** evidence hierarchy, gut microbiome, non-nutritive sweeteners, sucralose, tumor immunity

## Abstract

Non-nutritive sweeteners (NNS) are widely used sugar substitutes and constitute heterogeneous dietary and non-dietary exposures. Human gut microbiota findings vary by sweetener identity, purified compound versus commercial formulation, carrier ingredients, dose, duration, host background, and analytical method. Purified compounds and commercial formulations are therefore biologically non-equivalent. A structured narrative search of PubMed, Embase, and the Web of Science Core Collection was conducted from database inception through 17 July 2026 without language restrictions. Evidence was organized by directness to the clinical question and translational distance. Direct human evidence relating NNS exposure to immune checkpoint inhibitor (ICI) outcomes remains observational and sucralose-centered. Experimental evidence for gut microbiota involvement, arginine-related mediation, and reversibility derives mainly from preclinical models; comparable class-wide evidence is lacking. No prospective evidence currently supports NNS-specific dietary modification during immune checkpoint inhibitor therapy. Validation requires prospective, sweetener-specific exposure assessment integrated with gut microbiota, metabolomic, and immune profiling.

## 1 Introduction

Immune checkpoint inhibitors (ICIs) are a cornerstone of treatment for several solid tumors, and their efficacy is co-regulated by tumor-intrinsic characteristics, the tumor immune microenvironment, and host factors. Clinical and preclinical studies associate compositional and functional changes in gut microbial communities with ICI responsiveness, and fecal microbiota transplantation can transfer treatment-response phenotypes in experimental models (1–3). Antibiotics, proton pump inhibitors (PPIs), and dietary exposures may influence ICI efficacy and survival through gut microbiota remodeling and changes in microbial metabolic output (4, 5).

Non-nutritive sweeteners represent a host exposure of increasing interest. Evidence links selected NNS with gut microbiota and metabolic changes, but human effects remain inconsistent and their relevance to cancer immunotherapy is uncertain (6).

**Figure 1:**
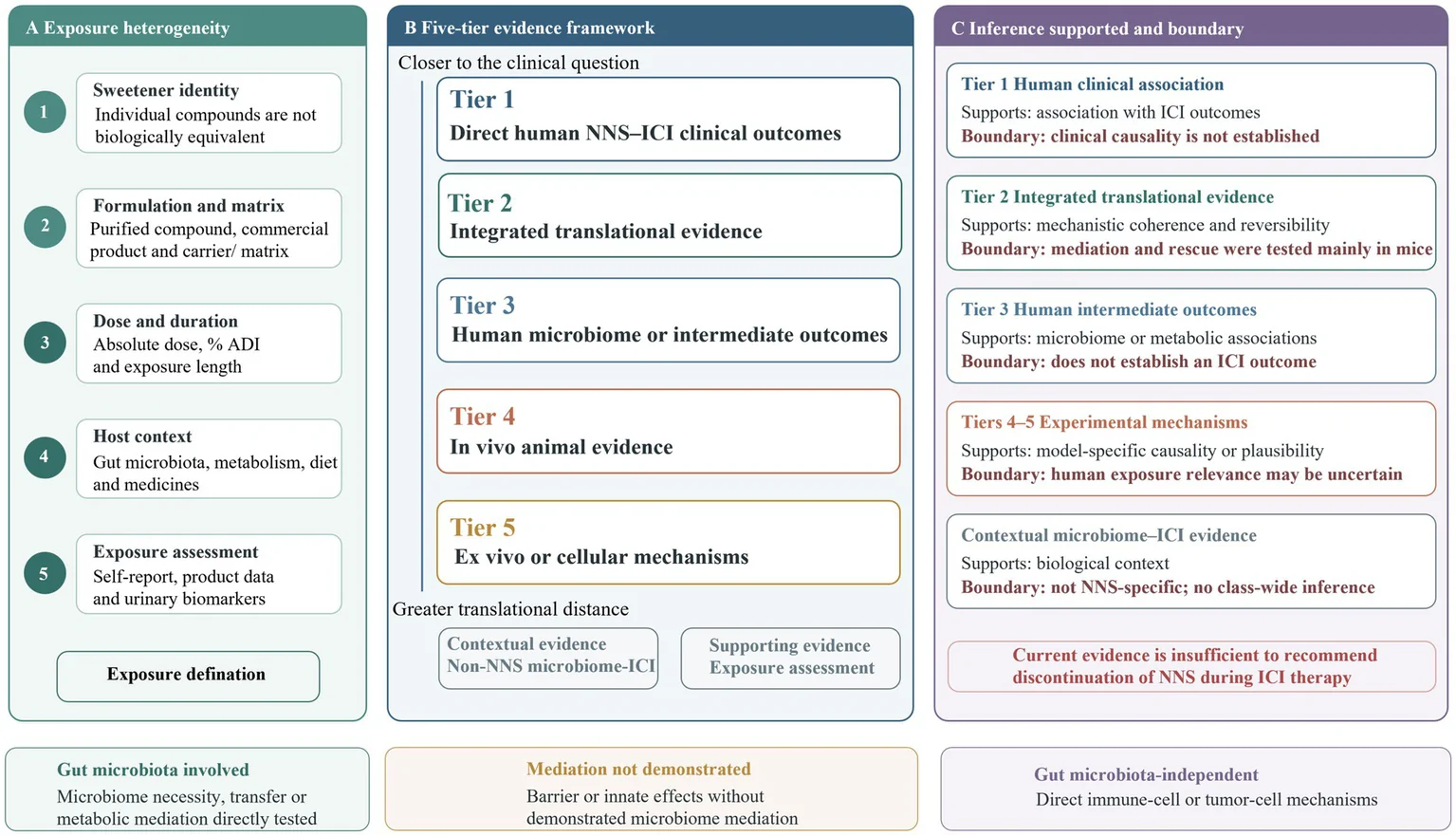
Five-tier evidence framework and mechanistic-route map for research linking non-nutritive sweeteners, the gut microbiome, and tumor immunity. **(A)** Summarizes the exposure dimensions required for biological interpretation: Sweetener identity, formulation and matrix, dose and duration, host context, and exposure assessment. **(B)** Presents the five-tier study-type hierarchy: Tier 1, direct human NNS–immune checkpoint inhibitor (ICI) clinical outcomes; Tier 2, integrated translational evidence linking human associations with experimental mediation or rescue; Tier 3, human microbiome or intermediate outcomes; Tier 4, *in vivo* animal evidence; and Tier 5, ex vivo or cellular mechanisms. **(C)** States the inference supported by each evidence source and its principal boundary. The lower band separately classifies the demonstrated relationship to the gut microbiota as gut microbiota involved, microbiota mediation not demonstrated, or gut microbiota-independent. These categories are orthogonal to Tier 1–5, which describe study type, proximity to the clinical question, and translational distance rather than certainty, study quality, or a formal evidence grade. ADI, acceptable daily intake; ICI, immune checkpoint inhibitor; NNS, non-nutritive sweetener.

This review synthesizes evidence linking NNS exposure, the gut microbiome, and tumor immunity while preserving distinctions among sweetener identity, formulation matrix, dose, duration, host characteristics, and endpoint type. The evidence framework is based on proximity to the clinical question and translational distance rather than a formal rating of study quality or certainty. Figure 1 separates exposure definition and the five-tier study-type hierarchy from three categories describing the relationship to the gut microbiota: gut microbiota involved, microbiota mediation not demonstrated, and gut microbiota-independent. Mechanistic relationship and evidentiary tier are orthogonal dimensions.

## 2 Methods

### 2.1 Literature search and study selection

A structured narrative literature search was conducted in PubMed, Embase, and the Web of Science Core Collection from database inception through 17 July 2026, without language restrictions. Three complementary search strategies addressed: (1) NNS exposure and the gut microbiome; (2) immune, inflammatory, epithelial-barrier, tumor-immunity, and immune checkpoint inhibitor-related effects of NNS; and (3) NNS exposure assessment, including dietary instruments, biomarkers, pharmacokinetics, and biological transfer. Controlled vocabulary and free-text terms for NNS and individual sweeteners were combined with microbiome-, immune-, barrier-, tumor-, immunotherapy-, and exposure-related terms. Reference lists of relevant studies and reviews were also examined to identify additional primary and contextual evidence.

After deduplication, records were assessed for relevance to the predefined scope. Eligible evidence included human interventional and observational studies, integrated translational studies, *in vivo* animal experiments, and ex vivo or cellular mechanistic studies in which an identifiable NNS exposure was related to microbiome, immune, epithelial-barrier, tumor, immunotherapy, or exposure-assessment outcomes.

### 2.2 Evidence framework

Reviews and guidelines were used for contextual synthesis and reference identification but were not treated as primary causal evidence. Evidence was organized as follows: Tier 1, direct human NNS–ICI clinical outcomes; Tier 2, integrated translational evidence linking human associations with experimental mediation or rescue; Tier 3, human microbiome or intermediate outcomes; Tier 4, *in vivo* animal evidence; and Tier 5, ex vivo or cellular mechanisms. Non-NNS microbiome–ICI studies were treated as contextual evidence, and exposure-assessment studies as supporting evidence. These categories indicate proximity to the clinical question and translational distance rather than a formal certainty-of-evidence rating.

## 3 NNS exposure: ubiquity, hidden sources, and measurement challenges

NNS confer sweetness while providing little or no energy and generally have greater sweetening intensity than sucrose. Synthetic NNS include acesulfame potassium, aspartame, cyclamate, saccharin, sucralose, neotame, and advantame; plant-derived NNS include steviol glycosides, monk fruit extracts, and thaumatin. Population data from NHANES 2009–2012 showed that 25.1% of children and 41.4% of adults consumed products containing NNS on a given day (7). Pharmaceutical formulations and oral-care products provide additional non-dietary sources, and studies of human milk, mother-to-infant transfer pharmacokinetics, amniotic fluid, cord blood, and environmental monitoring identify lactational, transplacental, and environmental exposure routes (8–12).

The 2023 WHO guideline uses the term “non-sugar sweeteners” and addresses their use for long-term weight control and reduction of noncommunicable-disease risk in the general population (13). The conditional recommendation is distinct from toxicological acceptable daily intakes and does not address cancer treatment or ICI efficacy. The sucralose–ICI association therefore neither validates nor extends the WHO recommendation. Both contexts support evaluation of overall dietary quality and habitual sweetness exposure rather than a single-product rule.

At the level of exposure assessment, existing studies have relied mainly on self-reported dietary tools, including food frequency questionnaires (FFQs), 24-h dietary recalls, and food records (14); in more refined dietary exposure estimation, these approaches may be supplemented with brand- or product-level information on sweetener concentrations. These methods can be used to estimate population intake, but they still depend on participant reporting and product concentration data, while actual sweetener levels in products may vary with blended use and product reformulation, and the degree to which individual sweeteners are distinguished is not consistent across studies.

Biomarker-based exposure assessment complements dietary self-report. Several commonly used NNS undergo limited metabolism and are excreted in urine, permitting measurement of recent exposure by liquid chromatography–tandem mass spectrometry (LC–MS/MS). The strongest urinary biomarker evidence covers acesulfame potassium, saccharin, sucralose, cyclamate, and steviol glucuronide as a marker of steviol glycosides. A validated LC–MS/MS method measures these five analytes, and a randomized double-blind crossover dose–response study showed discrimination among three short-term intake levels for acesulfame potassium, saccharin, cyclamate, and steviol glycosides using 24-h or fasting spot urine. Urinary sucralose discriminated low-dose intake (0.1% ADI) from high-dose intake (10% ADI) but not the intermediate level (15, 16).

Limited separation of intermediate sucralose doses does not establish a biological plateau. Sucralose has relatively low and variable fractional absorption, and urinary concentrations are influenced by collection timing, urine dilution, analytical variability, and between-person pharmacokinetics (17). At moderate habitual intakes, a single urinary measurement indicates recent exposure more reliably than continuous dose. Dose–response analyses require repeated 24-h or fasting spot samples, dilution correction, and product-level dietary records.

This strategy cannot be directly generalized to all NNS. The applicability of urinary intake biomarkers depends on whether the target sweetener enters urine as the parent compound or as a sufficiently specific metabolite; accordingly, direct urinary exposure biomarkers are not feasible for sweeteners such as aspartame, which undergo extensive hydrolysis and metabolism in the gastrointestinal tract. After ingestion, aspartame is primarily broken down into phenylalanine, aspartic acid, and methanol, and these common metabolites, rather than the intact molecule, enter the circulation, leaving no sufficiently specific urinary exposure marker (17). Accordingly, when evaluating associations between NNS and the gut microbiome, immune phenotypes, or immunotherapy-related outcomes, urinary biomarkers are better regarded as an objective complement for recent exposure to selected NNS rather than as a uniform measurement strategy applicable to all sweeteners.

## 4 NNS and the gut microbiome: heterogeneous findings and their sources

Preclinical studies have raised the possibility that selected NNS can alter microbial composition or function and thereby influence host metabolism. However, human evidence does not support a uniform class-wide microbiome effect across sweeteners, study designs, or endpoints.

Ceballos et al. synthesized literature linking excessive sugar and NNS exposure with reduced microbial diversity, proinflammatory microbial patterns, altered short-chain fatty acid production, and impaired epithelial-barrier integrity, but did not assess antitumor immunity or ICI outcomes (18). A human-focused review identified few controlled trials and substantial heterogeneity in NNS identity, formulation, exposure duration, and gut microbiota endpoints (19).

Human intervention studies have examined saccharin, sucralose, aspartame, stevia, and mixed beverage exposures, with findings ranging from null or limited overall microbiome effects in several purified-compound or short-term trials to detectable microbiome, metabolomic, or glycemic shifts in some saccharin- and sucralose-based studies (Table 1) (6, 20–29). Observational studies have also reported microbiome-associated signals in community-based adults, obesity cohorts, and maternal-exposure/infant-outcome settings (Table 2) (30–33). However, these designs are limited in their ability to establish directionality or causality, and NNS exposure is often estimated from self-reported intake rather than biomarker-validated measurement. Accordingly, the current evidence is better interpreted through a structured framework that first distinguishes exposure identity and formulation matrix, and only then considers dose, host responsiveness, exposure duration, and analytical endpoints.

**Table 1:** Interventional human studies of non-nutritive sweeteners, the gut microbiome, and intermediate outcomes.

Study	Design/population	*n*	NNS, formulation and carrier	Dose/Duration	Comparator/Exposure assessment	Microbiome method; direction and key quantitative results	Major limitations
Suez et al. (2014) (23)	Short intervention; healthy adults (Israel)	7	Saccharin; commercial formulation; carrier composition NR	5 mg/kg/day (33% standardized FDA ADI); 5–7 days	Pre–post comparisons; commercial product	16S: 4/7 participants developed poorer glycemic responses; responder-associated microbiome shifts were observed, and responder-derived FMT transferred glucose intolerance to germ-free mice.	Very small analysis set; commercial formulation; data-derived responder grouping; short exposure; no ICI outcomes
Thomson et al. (2019) (29)	Controlled intervention; healthy adults (Chile)	17	Sucralose capsule; calcium-carbonate carrier	780 mg/day (208% standardized FDA ADI); 7 days	Control capsules	16S: no significant change in glycemic or insulin outcomes, alpha/beta diversity, or taxon abundance; standardized effect estimates NR.	Small sample; 7-day exposure; microbiome outcomes exploratory; no ICI outcomes
Ahmad et al. (2020) (25)	Randomized double-blind crossover; healthy adults (Canada)	17	Pure sucralose in water; citric acid and lemon extract	136 mg/day (36% standardized FDA ADI); 14 days	Matched beverage/control period	16S: no change in Shannon diversity, abundant taxa, community structure (PERMANOVA *p* = 0.99), fecal SCFAs, or glycemic outcomes.	Small analysis set; 14-day exposure; microbiome was a secondary outcome; no ICI outcomes
Ahmad et al. (2020) (25)	Randomized double-blind crossover; healthy adults (Canada)	17	Pure aspartame in water; citric acid and lemon extract	425 mg/day (11% standardized FDA ADI); 14 days	Matched beverage/control period	16S: no change in Shannon diversity, abundant taxa, community structure (PERMANOVA *p* = 0.99), fecal SCFAs, or glycemic outcomes.	Small analysis set; 14-day exposure; microbiome was a secondary outcome; no ICI outcomes
Serrano et al. (2021) (27)	Randomized placebo-controlled intervention; healthy adults (United States)	13	Sodium saccharin capsule	400 mg/day (36% standardized FDA ADI); 14 days	Pulp-filler placebo capsule	16S: no treatment effect on alpha/beta diversity, taxon abundance, or OGTT; LEfSe identified no differentially regulated features.	Small analysis set; short exposure; lactisole arms not summarized here; no ICI outcomes
Méndez-García et al. (2022) (21)	Controlled intervention; healthy young adults (Mexico)	20	Pure sucralose in 60 mL sterile water	48 mg/day (13% standardized FDA ADI); 10 weeks	60 mL sterile-water control	qPCR: *Blautia coccoides* increased 3-fold and *Lactobacillus acidophilus* decreased to 0.66-fold versus controls; within the sucralose arm, insulin peak increased 32 ± 3.5% (*p* < 0.001) and glucose AUC increased 8 ± 1.7% (*p* = 0.02).	Open-label; small sample; qPCR limited community resolution; several metabolic comparisons were within-arm; no ICI outcomes
Suez et al. (2022) (6)	Randomized controlled trial; healthy adults (Israel)	20	Saccharin; commercial sachets with dextrose	16% standardized FDA ADI; 14 days	Dextrose-vehicle and untreated controls	Shotgun metagenomics: distinct stool/oral microbiome and plasma-metabolome changes; saccharin impaired group-level glycemic responses; standardized effect estimate NR.	Dextrose carrier; short exposure; personalized responder analyses; no ICI outcomes
Suez et al. (2022) (6)	Randomized controlled trial; healthy adults (Israel)	20	Sucralose; commercial sachets with dextrose	27% standardized FDA ADI; 14 days	Dextrose-vehicle and untreated controls	Shotgun metagenomics: distinct stool/oral microbiome and plasma-metabolome changes; sucralose impaired group-level glycemic responses; standardized effect estimate NR.	Dextrose carrier; short exposure; personalized responder analyses; no ICI outcomes
Suez et al. (2022) (6)	Randomized controlled trial; healthy adults (Israel)	20	Aspartame; commercial sachets with dextrose	6% standardized FDA ADI; 14 days	Dextrose-vehicle and untreated controls	Shotgun metagenomics: distinct microbiome/metabolome changes; no uniform group-level glycemic impairment, although responder-specific changes were reported; effect estimate NR.	Dextrose carrier; short exposure; responder analyses complicate uniform inference; no ICI outcomes
Suez et al. (2022) (6)	Randomized controlled trial; healthy adults (Israel)	20	Stevia; commercial sachets with dextrose	60% of the study-specified ADI; 14 days	Dextrose-vehicle and untreated controls	Shotgun metagenomics: distinct microbiome/metabolome changes; no uniform group-level glycemic impairment, although responder-specific changes were reported; effect estimate NR.	Dextrose carrier; steviol-equivalent conversion; short exposure; no ICI outcomes
Singh et al. (2024) (28)	Randomized open-label intervention; healthy adults (United Kingdom)	14	SteviaClear Sweet Drops; stevia extract and water	10 drops/day; active-glycoside dose NR; 12 weeks	No-intervention control (*n* = 13)	16S: no treatment effect on beta diversity (PERMANOVA *p* = 0.978) or Shannon diversity (*p* = 0.652), and no clear taxon-level differences; exploratory random-forest classification accuracy was 75%.	Small sample; open label; chemically quantified steviol-glycoside dose NR; product-level exposure; no ICI outcomes
Kwok et al. (2024) (26)	Randomized double-blind beverage trial; healthy adults (Canada)	27	Steviol-glycoside beverage; citric acid, potassium citrate, color and flavor	25% of the study-specified ADI; 4 weeks	30 g/day sucrose beverage (*n* = 32)	Shotgun metagenomics: no between-group differences in microbiome taxa or fecal SCFAs at week 4 (all *p* > 0.05); BMI was 0.3 kg/m^2^ higher with sucrose than stevia (*p* = 0.013).	Small arms; comparator differed in energy content; no ICI outcomes
Sylvetsky et al. (2024) (24)	Pilot intervention; healthy adults (United States)	17	Diet Rite Cola; sucralose + Ace-K	54% sucralose and 11% Ace-K standardized ADI; 1 week	Baseline/comparison period	Shotgun metagenomics: species-level relative-abundance shifts occurred after exposure; a community-wide standardized effect estimate was NR; metabolic outcomes NR.	Small pilot; mixed sweeteners and beverage matrix; no untreated parallel control; no ICI outcomes
Sylvetsky et al. (2024) (24)	Pilot intervention; healthy adults (United States)	8	Diet Pepsi; sucralose + Ace-K	19% sucralose and 11% Ace-K standardized ADI; 8 weeks	Baseline/comparison period	Shotgun metagenomics: species-level relative-abundance shifts occurred after exposure; a community-wide standardized effect estimate was NR; metabolic outcomes NR.	Very small pilot; mixed sweeteners and beverage matrix; no untreated parallel control; no ICI outcomes
Haslam et al. (2025) (20); type 2 diabetes	Randomized sugar-replacement trial; adults with type 2 diabetes (India)	49	Sucralose replacing sugar in coffee/tea	Mean approximately 13 mg/day; 12 weeks	Usual sugar use/control assignment	16S: alpha diversity decreased (Shannon difference −0.83, 95% CI − 1.49 to −0.17; Simpson −0.08, 95% CI − 0.16 to −0.01); beta-diversity group×time *p* = 0.007; 14 Firmicutes genera decreased and 2 increased (*q* < 0.20).	Low variable dose; reduction of sugar cannot be separated from addition of sucralose; self-reported adherence; genus-level data; no ICI outcomes
Haslam et al. (2025) (20); overweight/obesity	Randomized sugar-replacement trial; adults with overweight/obesity (India)	48	Sucralose replacing sugar in coffee/tea	Mean approximately 13 mg/day; 12 weeks	Usual sugar use/control assignment	16S: no overall beta-diversity treatment effect (group×time *p* = 0.50) and all alpha-diversity between-group *p* > 0.05; two genera met the exploratory FDR threshold (q < 0.20).	Low variable dose; dietary-fiber imbalance; self-reported adherence; subgroup-specific analysis; no ICI outcomes
Romo-Romo et al. (2025) (22)	Randomized placebo-controlled triple-blind trial; healthy adults (Mexico)	19	Pure sucralose capsule	270 mg/day planned; mean 252.9 mg/day (4.13 mg/kg/day; 72% standardized FDA ADI); 30 days	Identical cornstarch placebo capsule	16S: alpha diversity decreased (*p* < 0.0001). Glucose iAUC increased 132.1%, insulin iAUC 23.4%, and GLP-1 iAUC 67.6%; Matsuda index fell 24.1% versus 3.8% with placebo (between-group *p* = 0.02).	Small sample; 30-day exposure; several outcomes; no ICI outcomes

Ace-K, acesulfame potassium; ADI, acceptable daily intake; AUC, area under the curve; CI, confidence interval; FMT, fecal microbiota transfer; GLP-1, glucagon-like peptide 1; iAUC, incremental area under the curve; NR, not reported; OGTT, oral glucose tolerance test; SCFA, short-chain fatty acid; 16S, 16S rRNA gene amplicon sequencing; qPCR, quantitative polymerase chain reaction. Directional statements refer to the reported comparison specified in each row. Analyzed *n* refers to the listed exposure arm, substudy, or analysis set; comparator-group sizes are shown where they materially clarify interpretation and otherwise remain available in the primary study. All table cells report the direction of the finding and key quantitative results when available; NR is used when an effect estimate, dose, carrier composition, or endpoint was not reported. * Except where identified as study-specified, %ADI values were standardized for cross-study comparison using the US Food and Drug Administration definition and a 75-kg reference body weight. Steviol glycosides require conversion to steviol equivalents. FDA- and JECFA-based percentages are not interchangeable; agency-specific values and conversion rules are summarized in Supplementary Table S1. When the primary article lacked sufficient dose information, the value is NR. † In Suez et al. (6), saccharin and sucralose impaired glycemic responses at group level, whereas aspartame- and stevia-associated metabolic changes were mainly responder specific. § Romo-Romo et al. reported approximately 29.5–30% of the JECFA ADI (15 mg/kg/day) using mean body weight 61.0 ± 5.7 kg; the standardized FDA/75-kg value is 72%. These interventional studies inform microbiome and metabolic plausibility but did not directly evaluate tumor immunity or ICI outcomes.

**Table 2:** Observational human studies assessing non-nutritive sweetener exposure and the gut microbiome.

Study	Design/Population	*n*	Exposure definition	Exposure assessment	Microbiome method; direction and key quantitative results	Major limitations
Frankenfeld et al. (2015) (31)	Cross-sectional; US adults	31	Recent aspartame and/or Ace-K intake	4-day food record; 7 aspartame consumers, 7 Ace-K consumers, 3 overlapping	16S/pyrosequencing: no class/order abundance or predicted-function differences; overall diversity differed for aspartame consumers (*p* < 0.01) and Ace-K consumers (*p* = 0.03) versus nonconsumers.	Very small consumer groups; single stool sample; cross-sectional design; predicted functions; residual dietary confounding; no ICI outcomes
Farup et al. (2019) (30)	Cross-sectional; adults with morbid obesity (Norway)	89	Any NNS; individual compounds unresolved	Food-frequency questionnaire; 1 unit = 100 mL NNS beverage or 2 tablets/teaspoons; median 3.2 units/day (range 0–43)	GA-map: NNS intake was positively associated with Dysbiosis Index (B = 0.049, 95% CI 0.022–0.077; *p* = 0.001) and 4/39 bacterial groups; fecal butyrate was lower (partial *r* = −0.274; *p* = 0.011); no significant microbiota-mediated indirect effect.	Disease-specific cohort; compound and dose unresolved; many tests; cross-sectional design; residual confounding; no ICI outcomes
Ramne et al. (2021) (33)	Population-based cross-sectional study; Swedish adults	1,085	Artificially sweetened beverages (ASB); individual sweeteners unresolved	4-day food record and short FFQ	16S: several genera were nominally associated with ASB intake, but no ASB–microbiome association remained significant after multiple-testing correction; corrected signal was therefore null.	Exploratory cross-sectional analysis; beverage mixture; modest intake range; residual confounding; no ICI outcomes
Laforest-Lapointe et al. (2021) (32)	Prospective pregnancy–infant cohort (Canada)	100 infants	Maternal daily ASB exposure versus nonconsumption during pregnancy	Maternal food-frequency questionnaire; 50 exposed and 50 unexposed dyads	Infant-stool 16S: maternal ASB intake was associated with community-level shifts and depletion of several Bacteroides species; urinary succinate statistically mediated 29% of the association with BMI at 1 year.	Maternal exposure and infant microbiome measured in different individuals; selected substudy; individual sweeteners unresolved; residual confounding; no ICI outcomes

Ace-K, acesulfame potassium; ASB, artificially sweetened beverage; CI, confidence interval; FFQ, food-frequency questionnaire; NNS, non-nutritive sweetener; NR, not reported; 16S, 16S rRNA gene amplicon sequencing. For Laforest-Lapointe et al., exposure refers to maternal intake during pregnancy, whereas microbiome profiling was performed on infant stool samples. For Farup et al., microbiome profiling was performed using the GA-map Dysbiosis Test, not conventional 16S amplicon sequencing. These observational studies should be interpreted as association-level evidence only; most did not distinguish individual sweeteners with sufficient resolution and none directly assessed antitumor immunity or ICI response.

The evidence base is unevenly distributed across individual sweeteners and study endpoints. Interventional human studies have evaluated saccharin, sucralose, aspartame, steviol glycosides, and mixed sucralose/Ace-K beverage exposures, but the number of studies, formulations, exposure durations, and analytical methods differ substantially across sweeteners (Table 1). Sucralose has been examined more extensively than several other NNS, whereas evidence for aspartame, steviol glycosides, Ace-K, and mixed commercial products remains more limited in relation to immune-relevant outcomes. Several less-studied NNS, including neotame, advantame, and monk fruit-derived sweeteners, remain sparsely represented in controlled human microbiome studies. Collectively, the available studies therefore define a heterogeneous and uneven microbiome and metabolic evidence base rather than providing comparable evidence across individual NNS. Observational studies provide additional association-level signals, but they are limited by exposure misclassification, mixed-product intake, residual confounding, and the frequent inability to distinguish individual sweeteners (Table 2).

### 4.1 Exposure identity, formulation matrix, and dose comparability

The first interpretive issue is exposure identity rather than dose alone. Studies grouped under “NNS interventions” do not always test the sweetener molecule in isolation. Some human trials administered purified compounds in capsules or aqueous preparations, whereas others used commercial sachets, tabletop products, beverages, or mixed formulations containing additional carriers, bulking agents, or carbohydrates. When commercial preparations contain glucose, dextrose, maltodextrin, or other co-formulated ingredients, the biological exposure is more accurately understood as a formulation or matrix exposure rather than a sweetener-only exposure.

Formulation composition directly affects causal attribution. In the randomized trial by Suez et al., participants received saccharin, sucralose, aspartame, or stevia in sachet form, with a sachet-contained glucose vehicle control (6). In the earlier Suez et al. study, commercial saccharin, sucralose, and aspartame formulations contained approximately 5% sweetener and 95% glucose (23). The Splenda preparation evaluated in a preclinical study also contained dextrose and maltodextrin (34). Without an adequate vehicle-matched control, observed gut microbiota, metabolomic, or glycemic changes cannot be attributed specifically to the NNS molecule.

The human literature does not separate into simple positive and negative groups. Ahmad et al. reported no measurable gut microbiota or fecal short-chain fatty acid changes after 14 days of purified aspartame or sucralose, and Serrano et al. found no overall gut microbiota or glucose-tolerance effect after 14 days of purified saccharin at the maximal accepted intake (25, 27). Suez et al. reported gut microbiota, metabolomic, and glycemic changes after sachet-based exposures below the ADI, whereas Méndez-García et al. and Romo-Romo et al. reported gut microbiota-associated metabolic changes after longer sucralose exposure (6, 21, 22). These differences do not establish a carrier effect but leave sweetener-specific attribution unresolved when formulation and vehicle effects are not adequately controlled.

Dose comparability depends on formulation, absolute intake, ADI anchoring, and exposure duration. Fixed-dose supplementation with single sweeteners in preclinical studies and some human trials does not fully represent habitual exposure, which can involve lower doses, mixed food matrices, and products containing multiple sweeteners. Exposure definitions in NNS–gut microbiota research therefore require sweetener identity, formulation matrix, carrier composition, absolute dose, percentage of ADI, and duration. Agency-specific ADI values and the conversion rules applied in this review are summarized in Supplementary Table S1.

An *in vitro* screen tested 39 sweetener compounds against 25 phylogenetically diverse gut bacterial strains. Thirty reproducible sweetener–bacterium growth interactions were identified, and screening with four co-consumed compounds identified 102 combination effects, including 68 antagonistic and 34 synergistic interactions (35). Because the experiments used a single culture medium and an initial concentration of 50 μM, these findings demonstrate strain- and co-exposure-dependent microbial responses but do not establish *in vivo* or clinical effects.

### 4.2 Interindividual responsiveness and baseline gut microbiota

After formulation-related non-equivalence is considered, residual heterogeneity may reflect host-dependent response structure. Suez et al. observed personalized glycemic responses to different sweeteners, and microbiota from top and bottom responders transferred corresponding directional phenotypes to gnotobiotic mice (6). Average group-level effects can therefore obscure responder and non-responder patterns. In a nominally null trial, Thomson et al. found no overall effect of 7 days of high-dose sucralose on glycemic control or gut microbiota; however, gut microbiota composition differed between participants stratified by change in insulinemic response, independently of treatment allocation (29).

Suez et al. linked responder status to multivariable microbial and functional features, but the analysis did not identify a validated baseline taxon or pathway (6). Responder selection emphasized extremes of the glycemic distribution, feature sets differed by sweetener, and many signals emerged during exposure rather than forming a reproducible pretreatment signature. Predictive study designs require prespecified endpoints and models, absolute and relative abundance measurements, integration of metagenomic and metabolomic features with formulation and diet, and external validation before a taxon can be designated a responder biomarker.

Host genetics may provide an additional source of heterogeneity. TAS1R2 and TAS1R3 encode the heterodimeric sweet-taste receptor engaged by multiple NNS, and functional variation in TAS1R2 can alter receptor availability and postprandial glucose excursions (36). No study has established that TAS1R2/TAS1R3 genotype predicts NNS-induced gut microbiota changes, barrier effects, or ICI outcomes. Genotype-by-compound and genotype-by-formulation interactions remain testable hypotheses rather than established effect modifiers.

Observational studies in pregnancy–infant cohorts and adults with obesity or habitual sweetener intake identify settings in which gut microbiota-associated signals may differ by host background (30–33). Residual confounding from diet quality, metabolic status, medication exposure, and co-consumption of other sweeteners or food additives remains substantial. Host responsiveness therefore represents a second layer of heterogeneity after exposure identity and formulation.

### 4.3 Sex as a source of response heterogeneity

Sex can shape immune tone, hormone–microbiota interactions, body composition, and dietary exposure patterns. In a large multi-ethnic human cohort, sex and menopausal status showed modest associations with gut microbiota composition and selected functional pathways, with partial attenuation after adjustment for diet and other covariates (37). Current human NNS interventions remain too small to estimate sex-by-exposure interactions reliably.

Sex-related heterogeneity in ICI efficacy has been reported in a systematic review and meta-analysis (38), whereas its relevance to NNS exposure remains unknown. Priority NNS study designs include sex-disaggregated enrollment and outcomes, separation of biological sex from gender-related behaviors, documentation of menopausal and hormonal status, and prespecified interaction analyses.

### 4.4 Exposure duration, analytical resolution, and endpoint hierarchy

Signal detection depends on exposure duration and analytical strategy. Most human intervention studies used 7–14-day supplementation. Null findings after short-term exposure to purified aspartame, sucralose, or saccharin neither exclude biological effects nor establish inadequate analytical sensitivity (25, 27). Detectable responses vary with compound identity, formulation matrix, exposure intensity, host background, and endpoint.

General perturbation studies provide temporal context for NNS research: diet altered gut microbiota structure within days (39), whereas recovery after antibiotic exposure remained incomplete or taxon-specific for months (40). NNS-specific recovery kinetics have not been established. A 7–14-day null result may reflect insufficient induction time, whereas an end-of-intervention difference may represent a transient perturbation. Priority trial designs include serial sampling during exposure, at least two post-cessation time points, documented washout adequacy, and separate assessment of taxonomic, functional, and metabolomic recovery.

Analytical resolution is equally important. Amplicon-based 16S sequencing mainly captures compositional information at the genus or family level and is well suited to describing community structure. However, when the dominant effect lies in microbial pathway activity or metabolite output, taxonomic shifts may remain modest or undetectable. Shotgun metagenomics provides higher resolution at the species and functional-gene levels, while metabolomics can capture downstream biochemical changes that may be more directly relevant to host metabolism or immune regulation (6, 23). Absence of a strong 16S-based taxonomic shift therefore does not exclude functional effects, particularly when metabolomic or glycemic endpoints show changes.

A 2026 dynamic colonic simulator study using child-derived human gut microbiota reported dose-dependent taxonomic, functional, and metabolic responses to acesulfame potassium, including microbial degradation pathways, altered short-chain fatty acid production, and reduced Caco-2 epithelial integrity after exposure to simulator effluent (41). This ex vivo human-microbiota model provides mechanistic evidence rather than a clinical barrier outcome.

NNS–gut microbiota findings depend jointly on sweetener identity, formulation matrix, carrier composition, absolute dose and ADI anchoring, baseline gut microbiota, host metabolic state, exposure duration, and endpoint layer. Informative human designs separate purified compounds from commercial products, use vehicle-matched controls, report absolute dose and percentage of ADI, control dietary background, prespecify responder analyses, and combine compositional profiling with metagenomics, metabolomics, and relevant clinical phenotypes.

### 4.5 Diversity–function relationships

Alpha diversity describes within-sample richness or evenness, whereas beta diversity describes between-sample dissimilarity; neither directly quantifies metabolic output (42). Similar diversity profiles can coexist with different pathway or metabolite profiles, and taxonomic changes may not produce proportional functional changes. Diversity indices therefore characterize community structure rather than uniformly beneficial or harmful states.

An integrated NNS study design pairs alpha and beta diversity with species- or strain-resolved metagenomics, pathway abundance or expression, absolute microbial load, targeted metabolomics, and host immune phenotypes. This design separates structural community changes from functional effects and avoids treating modest diversity loss as evidence of impaired antitumor immunity.

## 5 NNS-related immune mechanisms by demonstrated relationship to the gut microbiota

Effective antitumor immunity depends on T-cell activation, persistence and metabolic fitness, antigen recognition, nutrient availability, epithelial-barrier integrity, and controlled inflammatory signaling. The NNS literature does not establish a single linear gut microbiota-mediated pathway. Available evidence comprises immunometabolic findings involving gut microbiota, barrier and innate immune findings without demonstrated microbiota mediation, direct immune-cell effects, and tumor-intrinsic mechanisms. These categories describe the demonstrated relationship to the gut microbiota, whereas the evidence tiers describe study type and translational distance.

### 5.1 Immunometabolic findings involving gut microbiota

Selected NNS alter microbial composition or function in preclinical models, but the exposure conditions differ materially. In Guo et al., male C57BL/6 mice received sucralose at 1.5 mg/mL ad libitum in drinking water for 6 weeks, followed by 2.5% dextran sulfate sodium (DSS) for 7 days; plain drinking water/DSS served as comparator, one sucralose concentration was tested, and dose–response evaluation was NR (43). In Li et al., mice received 1.5 mg/mL sucralose in drinking water for 6 weeks before azoxymethane (10 mg/kg intraperitoneally) and continued exposure during two 5-day 2.5% DSS cycles; the azoxymethane/DSS group without sucralose was the vehicle-matched comparator, one concentration was tested, and tumor incidence was 87.5% versus 50% (44). In Bian et al., mice received saccharin at 0.3 mg/mL in drinking water for 6 months with plain-water controls; one concentration was tested and dose–response evaluation was NR (45). Conversion of these drinking-water concentrations to mg/kg/day or %ADI was NR because individual water intake and time-resolved body weight were not reported in a form permitting reliable calculation. These disease-induction models do not represent habitual human exposure or ICI-treated cancer.

Zhu et al. exposed Apc^min/+^ mice to 0, 0.07, or 0.2 mg/mL aspartame in drinking water for 3 months, providing a water control and two active concentrations (46). Both concentrations accelerated intestinal tumor progression, accompanied by enrichment of *Akkermansia muciniphila* and increased microbial 5-hydroxytryptophan production. The two-dose design provides evidence of concentration-related evaluation, but a quantitative dose–response slope and mg/kg/day exposure were NR. Direct conversion to %ADI was also NR because water intake and longitudinal body weight were insufficient for a reliable calculation. The model evaluates intestinal tumorigenesis rather than antitumor immunity or ICI efficacy.

**Figure 2:**
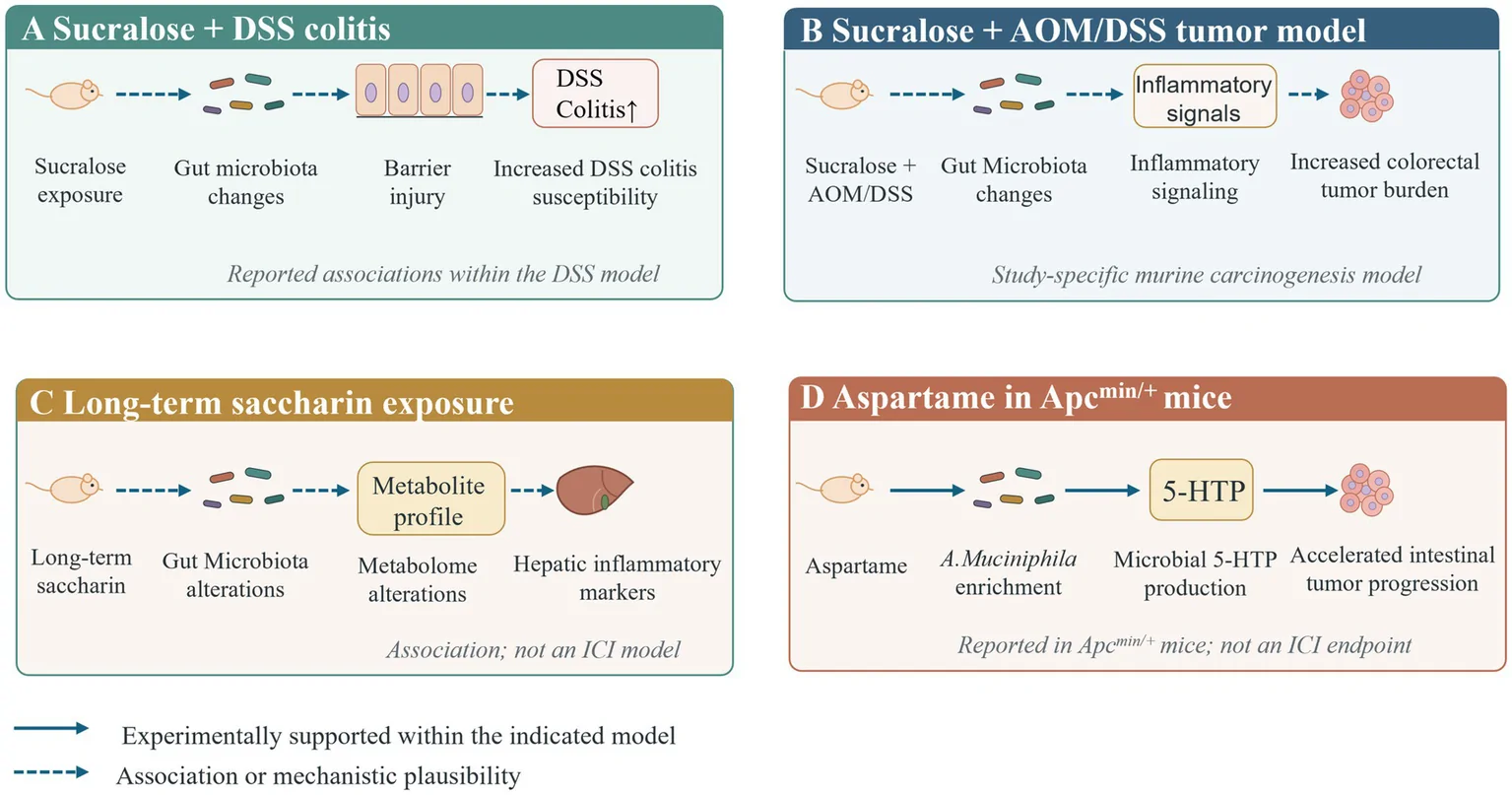
Selected preclinical findings involving gut microbiota. Four distinct study-specific mouse models are presented in parallel. **(A)** In a dextran sulfate sodium (DSS) colitis model, sucralose increased colitis susceptibility in association with gut microbiota changes and epithelial-barrier injury. **(B)** In an azoxymethane/DSS model of colitis-associated colorectal carcinogenesis, sucralose exposure was accompanied by gut microbiota changes, inflammatory signaling, and increased colorectal tumor burden. **(C)** Long-term saccharin exposure was associated with gut microbiota and metabolome alterations together with hepatic inflammatory markers. **(D)** In Apc^min/+ mice, aspartame exposure was linked to enrichment of *Akkermansia muciniphila*, increased microbial 5-hydroxytryptophan production, and accelerated intestinal tumor progression. Solid arrows indicate experimentally supported relationships within the indicated model, whereas dashed arrows indicate reported associations or mechanistic plausibility. The studies used different exposure conditions and disease models and did not evaluate ICI outcomes or habitual human exposure. AOM, azoxymethane; DSS, dextran sulfate sodium; 5-HTP, 5-hydroxytryptophan; ICI, immune checkpoint inhibitor.

Microbiota-derived metabolites provide an intermediate layer between NNS-associated ecological perturbation and immune regulation. Evidence linking microbial metabolites or nutrient-availability pathways to antitumor T-cell function derives mainly from broader microbiome–immunity studies rather than NNS-specific experiments. These mechanisms provide biological context but do not establish an NNS-induced effect on tumor immunity or ICI response. Figure 2 presents the available findings as parallel, study-specific preclinical models.

Microbiota-derived products can influence distal tumors through several non-exclusive routes. After intestinal production, absorbable metabolites enter portal blood, may be transformed by the intestinal epithelium and liver, and can reach the systemic circulation; they may then act directly within tumor interstitial fluid or indirectly by programming circulating or lymphoid immune cells before tumor entry. Inosine provides a proof-of-principle in which a microbiota-derived metabolite enhanced checkpoint-blockade efficacy and T-cell function in mice (47). For most candidate NNS-linked metabolites—including short-chain fatty acids and amino-acid pools—paired measurements in stool, portal or peripheral blood, tumor tissue, and tumor interstitial fluid are lacking. A systemic association alone does not establish a biologically active intratumoral concentration.

Tumor-type-specific intracellular bacteria have been detected in human tumors and immune cells (48). Whether tumor-resident microorganisms generate metabolites that modify local immunity, or whether NNS exposure alters these microorganisms, remains unknown. No NNS-specific evidence demonstrates microbial translocation from gut to tumor. Direct evaluation requires isotope tracing, spatial metabolomics, paired stool–plasma–tumor sampling, and low-biomass contamination controls.

### 5.2 Gut microbiota-independent T-cell-intrinsic effects of sucralose

Gut microbiota-independent T-cell effects have been evaluated most directly for sucralose. Zani et al. administered sucralose ad libitum in drinking water at 0.17 or 0.72 mg/mL; plain water was the vehicle control, and tumor and infection cohorts were exposed for 2 weeks before challenge and throughout follow-up (49). Both doses were evaluated *in vivo*, and sucralose inhibited T-cell responses dose-dependently *in vitro*; sodium saccharin and acesulfame potassium comparators did not reproduce a comparable degree of suppression. Under the authors’ 10-week body-surface-area scaling, 0.17 mg/mL approximated the FDA ADI and 0.72 mg/mL approximated the EFSA ADI. These values are allometric experimental benchmarks rather than direct equivalents of habitual human intake. Sucralose reduced T-cell membrane order, TCR-proximal PLCγ1 activation, downstream calcium mobilization, proliferation, differentiation, and antigen-specific CD8+ T-cell responses.

Membrane biophysical and T-cell signaling studies show that altered membrane organization can influence immune-synapse stability, PLCγ1 activation, Ca^2+^ mobilization, and T-cell functional thresholds (50–53). These studies support the plausibility of the sucralose–T-cell mechanism but do not provide additional NNS-specific or gut microbiota-mediated tumor-immunity evidence.

**Figure 3:**
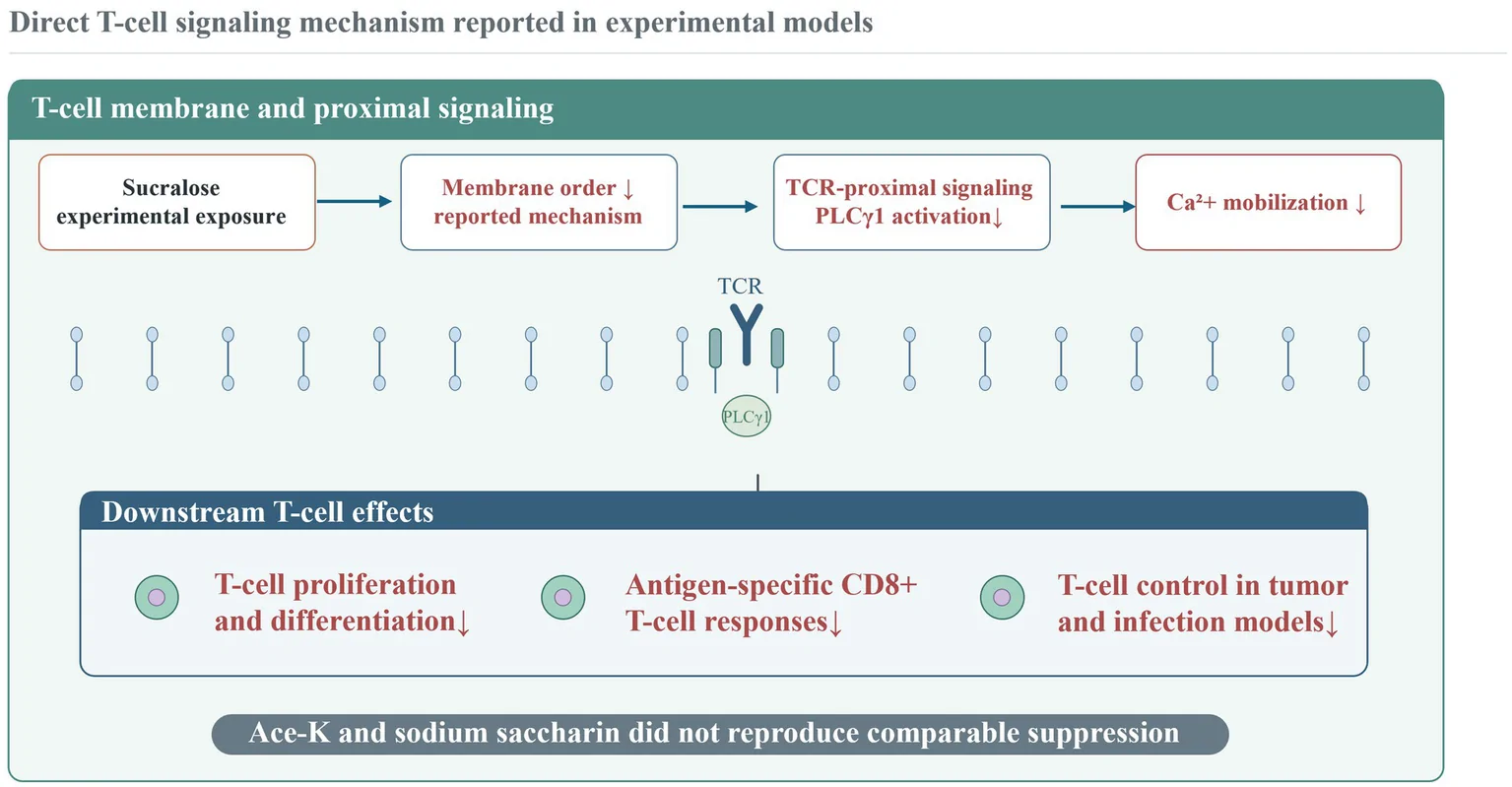
Gut microbiota-independent, T-cell-intrinsic effects of sucralose. In experimental models, sucralose reduced T-cell membrane order, T-cell receptor (TCR)-proximal PLCγ1 activation, and downstream Ca^2+^ mobilization, with reduced T-cell proliferation and differentiation, impaired antigen-specific CD8^+^ T-cell responses, and weaker T-cell-mediated control in tumor and infection models. Solid arrows indicate relationships reported within the same experimental framework. Membrane biophysical studies and broader literature linking membrane order, immune-synapse organization, PLCγ1 signaling, and T-cell function provide mechanistic context but not additional NNS-specific clinical evidence. Acesulfame potassium and sodium saccharin did not reproduce a comparable degree of T-cell suppression in the same experimental framework. The applicability of this compound-specific mechanism to habitual human exposure remains unestablished. Ace-K, acesulfame potassium; NNS, non-nutritive sweetener; PLCγ1, phospholipase C gamma 1; TCR, T-cell receptor.

Under the same experimental framework, acesulfame potassium and sodium saccharin did not reproduce a comparable degree of T-cell suppression. Direct T-cell inhibition is therefore a sucralose-related, gut microbiota-independent effect rather than a shared feature of all NNS. Figure 3 summarizes this compound-specific mechanism.

### 5.3 Barrier and innate immune mechanisms without demonstrated gut microbiota mediation

Barrier tissues and innate immune cells provide potential routes between NNS exposure and immune phenotypes, but the available studies did not demonstrate gut microbiota mediation. In Caco-2 epithelial models, cells were exposed to 0.1 mM sucralose, saccharin, or aspartame for 24 h; 0 μM vehicle and lipopolysaccharide at 1 μg/mL served as negative and positive controls, respectively (54). Sucralose and aspartame increased permeability through T1R3-related pathways, and T1R3 knockdown or claudin-3 overexpression mitigated selected phenotypes. Higher concentrations were evaluated for viability, apoptosis, and oxidative stress. Dose–response evidence was limited to selected endpoints, and comparison with an oral ADI is not physiologically meaningful for a cell-culture concentration.

A subsequent study extended aspartame-related findings across gastrointestinal epithelial cells, human intestinal organoids, and gut-on-a-chip models (55). Cytotoxicity was detected from 1.25 mg/mL after 24–48 h; organoids were exposed to 2.5 or 10 mg/mL for 48 h, and gut-on-a-chip transcriptomic experiments used 2.5 or 10 mg/mL for 24 h. Barrier impairment was concentration dependent, while oxidative-stress, inflammatory, and DNA-repair pathways were altered. Untreated controls were included; model-specific solvent composition was NR. Oral ADI comparison is not directly applicable to *in vitro* concentrations, and correspondence to luminal concentrations during habitual intake remains uncertain.

**Figure 4:**
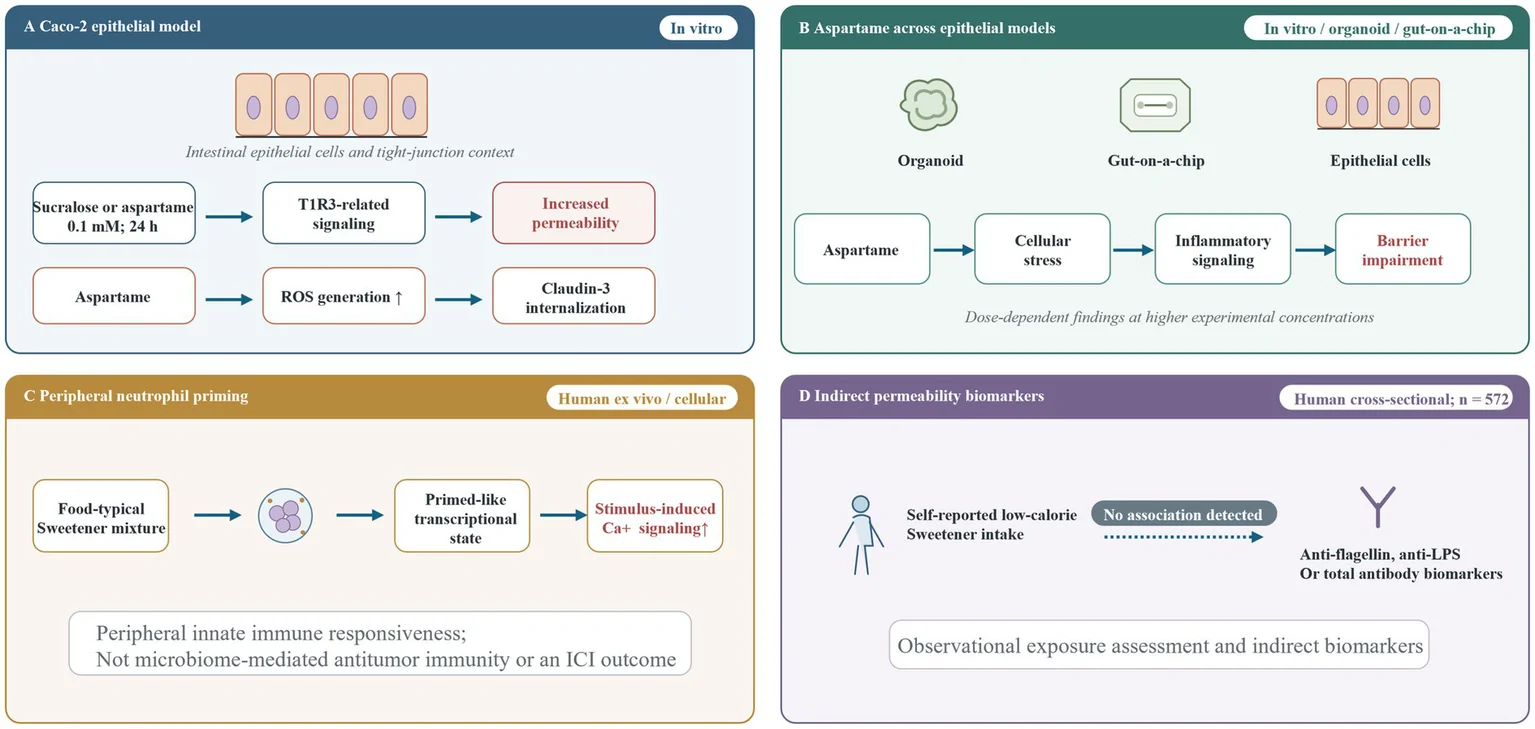
Barrier and innate immune findings without demonstrated gut microbiota mediation. **(A)** In Caco-2 epithelial models, 0.1 mM sucralose or aspartame increased permeability after 24 h through T1R3-related pathways. Aspartame-associated permeability was accompanied by reactive oxygen species generation and claudin-3 internalization. **(B)** A subsequent study across gastrointestinal epithelial cells, intestinal organoids, and gut-on-a-chip models reported aspartame-associated cellular stress, inflammatory signaling, and dose-dependent barrier impairment at higher experimental concentrations. **(C)** A food-typical sweetener mixture shifted neutrophil transcriptional profiles toward a primed-like state and enhanced stimulus-induced Ca^2+^ signaling. This finding reflects peripheral innate immune responsiveness rather than gut microbiota-mediated antitumor immunity. **(D)** In a cross-sectional analysis of 572 adults, self-reported NNS intake was not associated with circulating anti-flagellin, anti-lipopolysaccharide, or total antibody biomarkers used as indirect indicators of intestinal permeability. None of these studies demonstrated gut microbiota mediation or an effect on ICI outcomes. ICI, immune checkpoint inhibitor; LPS, lipopolysaccharide; NNS, non-nutritive sweetener; ROS, reactive oxygen species; T1R3, taste receptor type 1 member 3.

A randomized crossover pilot in 10 healthy adults used a single beverage containing sodium saccharin 75.6 mg/L, acesulfame potassium 52.7 mg/L, and sodium cyclamate 227.7 mg/L in mineral water (10.7 mL/kg body weight), with mineral water as the control and blood sampling at 0, 4, 8, and 24 h (56). *In vitro* neutrophils were pre-exposed for 10 min to a plasma-matched mixture of 1 μM cyclamate, 0.7 μM saccharin, and 0.3 μM acesulfame potassium before fMLF stimulation; a 30-fold mixture was also tested, with <0.1% dimethyl sulfoxide as the solvent control. The mixture shifted neutrophil transcriptional profiles toward priming and increased stimulus-induced calcium signaling. The study evaluated an acute mixture rather than an individual-compound dose–response relationship, and ADI-based attribution to any single sweetener is not possible. In a separate cross-sectional analysis of 572 US adults participating in the Cancer Prevention Study-3 Diet Assessment Substudy, average intake of aspartame, sucralose, acesulfame potassium, and saccharin was estimated from up to six 24-h dietary recalls collected over 12 months, while circulating anti-flagellin, anti-lipopolysaccharide, and total antibodies were measured in up to two fasting blood samples collected 6 months apart (57). Nonconsumers (*n* = 257; median intake, 0 mg/day) were compared with lower consumers (>0 to ≤50th percentile; *n* = 158; median intake, 11.3 mg/day) and higher consumers (>50th percentile; *n* = 157; median intake, 124.2 mg/day) using multivariable linear regression adjusted for sociodemographic characteristics, lifestyle factors, and medical history. Neither total nor individual sweetener intake was associated with any of the three permeability biomarkers; adjusted mean differences and 95% CIs were NR. The cross-sectional design, self-reported dietary recalls, combined exposure to multiple compounds, and use of indirect circulating biomarkers limit causal and compound-specific inference. Figure 4 compares barrier, innate immune, and population findings without demonstrated gut microbiota mediation.

### 5.4 Tumor-intrinsic immune-evasion pathways

Tumor-intrinsic evidence remains limited to cellular systems. In hepatocellular carcinoma cells, Kim et al. reported that acesulfame potassium increased PD-L1 protein without a corresponding increase in PD-L1 mRNA by suppressing autophagic degradation through ERK1/2–mTORC1 signaling, thereby reducing T cell-mediated tumor-cell killing (58). Concentration, exposure duration, vehicle, and quantitative dose–response data were NR in the accessible primary report. An oral ADI comparison is not applicable to the unreported *in vitro* concentration.

**Figure 5:**
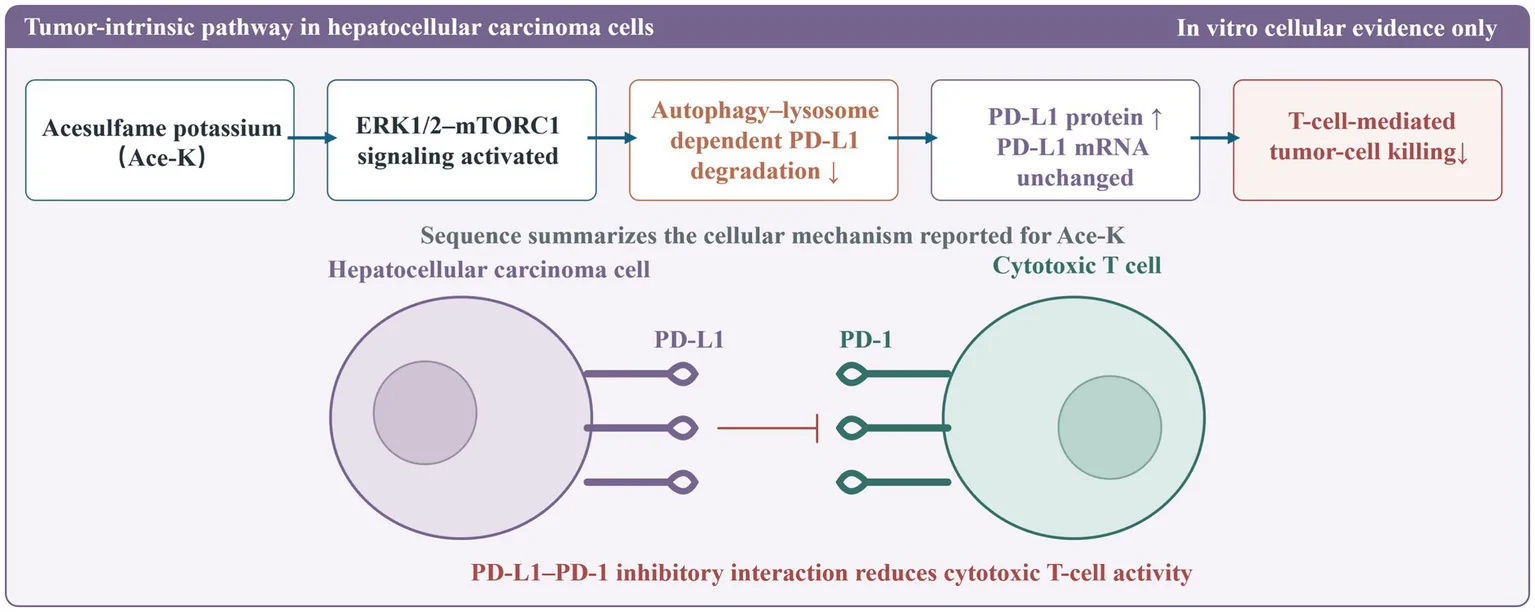
Tumor-intrinsic stabilization of programmed death-ligand 1 by acesulfame potassium in hepatocellular carcinoma cells. The numbered sequence summarizes the cellular mechanism reported for acesulfame potassium (Ace-K). Ace-K activated ERK1/2–mTORC1 signaling and attenuated autophagy–lysosome-dependent degradation of programmed death-ligand 1 (PD-L1), thereby increasing PD-L1 protein without a corresponding increase in PD-L1 mRNA. Increased tumor-cell PD-L1 strengthened the PD-L1–PD-1 inhibitory interaction and reduced T-cell-mediated tumor-cell killing in the cellular system. The red inhibitory bar denotes suppression of cytotoxic T-cell activity at the tumor-cell–T-cell interface. Broader tumor-biology literature supports autophagic regulation of PD-L1, but the Ace-K finding remains an *in vitro* mechanism without validated in vivo dose relevance or evidence of altered ICI outcomes. Ace-K, acesulfame potassium; ERK1/2, extracellular signal-regulated kinase 1/2; ICI, immune checkpoint inhibitor; mTORC1, mechanistic target of rapamycin complex 1; PD-1, programmed cell death protein 1; PD-L1, programmed death-ligand 1.

Because PD-L1 can be regulated post-translationally through autophagy–lysosome-dependent degradation (59), the acesulfame-K finding suggests a possible tumor-intrinsic immune-evasion route. However, it remains a cellular mechanism without validated *in vivo* exposure relevance or evidence of altered ICI outcomes. The tumor-intrinsic Ace-K–PD-L1 mechanism reported in hepatocellular carcinoma cells is depicted in Figure 5.

## 6 NNS and ICI response: current evidence centered on sucralose

Direct evidence relating NNS exposure to ICI outcomes is limited to a sucralose-centered observational and translational study. Evidence for other sweeteners remains indirect and derives from human intermediate outcomes or animal and cellular mechanisms. Biological plausibility, clinical association, and evidence sufficient to change patient management therefore remain distinct evidentiary claims.

### 6.1 Contextual microbiome–ICI evidence and its boundary

Microbiome–ICI studies provide biological context but do not demonstrate that NNS exposure alters ICI efficacy. Baseline microbial features, fecal microbiota transfer, and microbiome-targeted interventions can influence checkpoint-blockade response in selected cancer settings (60–62), while fiber-associated microbial functions, inosine, short-chain fatty acids, and amino-acid availability can affect T-cell differentiation and metabolic fitness (47, 63–65). These studies establish the biological relevance of the microbiome–immunity axis, but they are classified as contextual because they did not test NNS exposure.

### 6.2 Sucralose as a single-study translational model: clinical association and experimental support

The study by Morder et al. integrates patient associations with animal experiments, gut microbiota analyses, immunometabolic measurements, and rescue interventions (66). The patient data establish association, whereas gut microbiota involvement, arginine-related mediation, causal manipulation, and reversibility were evaluated mainly in mice.

Human clinical associations. The prospective registry analysis included 132 patients with advanced cancer—91 with melanoma and 41 with non-small cell lung cancer—who received anti–PD-1-based immunotherapy or chemoimmunotherapy (66). Dietary exposure was estimated using the web-based Diet History Questionnaire III, which was administered before systemic therapy. A cohort-derived threshold of >0.16 mg/kg/day defined higher sucralose intake. Higher intake was associated with a trend toward a lower objective response rate in the advanced melanoma cohort and a significantly lower objective response rate in the non-small cell lung cancer cohort, together with shorter progression-free survival in both cohorts. In advanced melanoma, higher intake was independently associated with a lower probability of objective response (multivariable OR, 0.28; 95% CI, 0.085–0.924; *p* = 0.0366) and poorer progression-free survival (univariable HR, 2.21; 95% CI, 1.02–4.79; *p* = 0.0449; multivariable HR, 2.91; 95% CI, 1.22–6.96; *p* = 0.0164). A separate cohort included 25 patients with high-risk resectable melanoma treated with neoadjuvant nivolumab plus the intratumoral TLR9 agonist vidutolimod; higher sucralose intake was associated with a lower major pathologic response rate and shorter relapse-free survival.

The clinical findings remain vulnerable to exposure misclassification because intake was self-reported rather than biomarker-validated. Dietary pattern, fiber intake, metabolic status, disease burden, concomitant medication, socioeconomic factors, and co-exposure to other sweeteners provide potential residual confounding. Additional limitations include the data-derived intake threshold, multiple outcomes and cohorts, limited tumor-type distribution, absence of independent prospective replication, and incomplete benchmarking against established predictors such as PD-L1 expression, tumor mutational burden, disease burden, and prior treatment context. The evidence therefore constitutes an observational signal rather than an established clinical mechanism.

**Figure 6:**
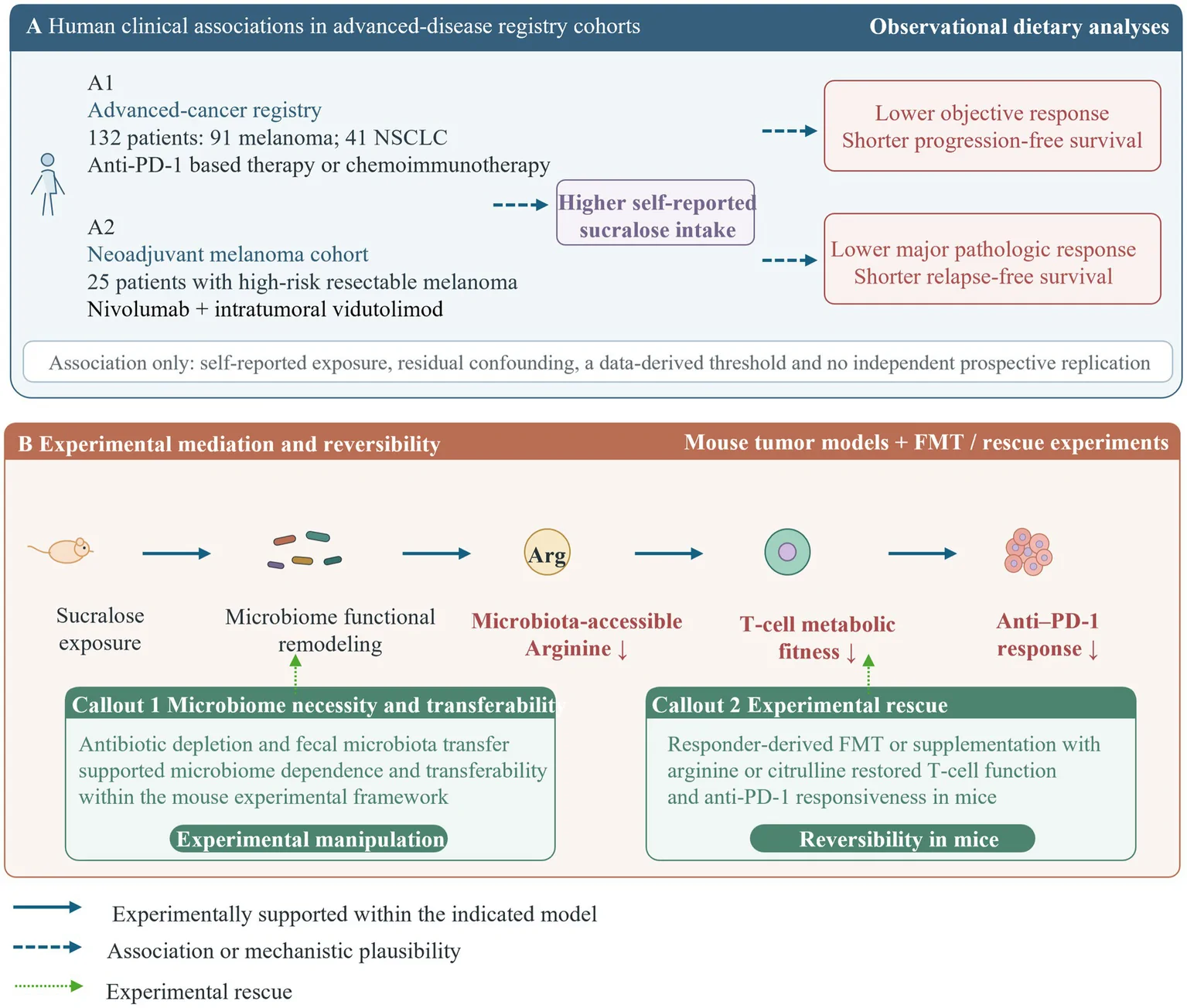
Sucralose-centered immune checkpoint inhibitor evidence: human clinical associations versus experimental mediation and reversibility. **(A)** Human clinical associations. **(A1)** A prospective registry analysis included 132 patients with advanced cancer—91 with melanoma and 41 with non-small cell lung cancer—who completed the web-based, semiquantitative Diet History Questionnaire III before receiving anti–PD-1-based immunotherapy or chemoimmunotherapy. Higher self-reported sucralose intake was defined using a cohort-derived threshold of >0.16 mg/kg/day. Higher intake was associated with a trend toward a lower objective response rate in the advanced melanoma cohort, a significantly lower objective response rate in the non-small cell lung cancer cohort, and shorter progression-free survival in both cohorts. In advanced melanoma, higher intake was independently associated with a lower probability of objective response (multivariable OR, 0.28; 95% CI, 0.085–0.924; *p* = 0.0366) and poorer progression-free survival (univariable HR, 2.21; 95% CI, 1.02–4.79; *p* = 0.0449; multivariable HR, 2.91; 95% CI, 1.22–6.96; *p* = 0.0164). **(A2)** A separate cohort included 25 patients with high-risk resectable melanoma treated with neoadjuvant nivolumab plus the intratumoral TLR9 agonist vidutolimod. Higher sucralose intake was associated with a lower major pathologic response rate and shorter relapse-free survival. The dietary analyses in both clinical settings were observational and remain susceptible to self-reported exposure misclassification, residual dietary and clinical confounding, the cohort-derived threshold, multiple outcomes and cohorts, limited tumor-type representation, and the absence of independent prospective replication. **(B)** Experimental mediation and reversibility. In mouse tumor models, sucralose was administered in drinking water at 0.09 mg/mL for 2 weeks before tumor implantation and continued during anti–PD-1 treatment. Antibiotic depletion and fecal microbiota transfer supported gut microbiota involvement and transferability. Sucralose-associated functional remodeling of the gut microbiota and reduced microbiota-accessible arginine were linked to impaired T-cell metabolic fitness and attenuated anti–PD-1 response. Responder-derived fecal microbiota transfer and supplementation with arginine or citrulline restored T-cell function and anti–PD-1 responsiveness. Blue dashed arrows in Panel A indicate observational human associations; blue solid arrows in Panel B indicate experimentally supported relationships within the mouse models; and green dotted arrows indicate experimental manipulation or rescue. The mouse experiments demonstrate preclinical mechanistic coherence and reversibility but do not establish clinical causality. CI, confidence interval; FMT, fecal microbiota transplantation; HR, hazard ratio; ICI, immune checkpoint inhibitor; MPR, major pathologic response; NSCLC, non-small cell lung cancer; OR, odds ratio; PD-1, programmed cell death protein 1; PFS, progression-free survival; RFS, relapse-free survival; TLR9, Toll-like receptor 9.

Experimental mediation and reversibility. In mouse tumor models, sucralose at 0.09 mg/mL in drinking water was administered for 2 weeks before tumor implantation and continued during anti–PD-1 treatment (66). Antibiotic depletion and fecal microbiota transfer supported gut microbiota involvement and transferability, whereas reduced microbiota-accessible arginine was linked to impaired T-cell metabolic fitness. Responder-derived fecal microbiota transfer and supplementation with arginine or citrulline restored T-cell function and anti–PD-1 responsiveness. These experiments demonstrate preclinical mechanistic coherence and rescue but do not establish clinical causality. Independent prospective cohorts and biomarker-supported exposure assessment are required for clinical validation. Figure 6 separates the observational human associations from the experimentally supported mouse mediator–rescue model.

### 6.3 Causal structure and cross-species transferability

Temporality determines the causal role of candidate variables in the sucralose–ICI association. Pre-exposure dietary quality, fiber intake, metabolic status, disease burden, concomitant medication, socioeconomic factors, and co-exposure to other sweeteners act as confounders when they influence both reported sucralose intake and ICI outcome; they become mediators only if sucralose exposure changes them. Post-exposure gut microbiota remodeling, altered arginine availability, and T-cell metabolic fitness are candidate mediators. Estimation of total and direct effects requires explicit causal diagrams, covariate selection that preserves true mediators, control of mediator–outcome confounding, and assessment of reverse causation from illness- or treatment-related changes in beverage choice.

Mouse experiments provide causal leverage but limited direct human generalizability. Murine gut microbiota, chow, housing, sweetener exposure, tumor implantation, and immune physiology differ from those of patients. Human microbiota transfer into germ-free mice can produce selective engraftment and metabolic divergence from the donor community (67). Translational validation requires donor-to-recipient engraftment data, replication across donors and facilities, concordant metabolite and immune changes in human samples, and dose validation in prospective oncology cohorts.

### 6.4 Clinical implications for ICI candidates

Current evidence does not support a universal instruction to discontinue sucralose or other NNS in patients receiving ICI therapy. The clinical signal is observational, is based mainly on one sucralose-centered study, and establishes neither a safe nor a harmful threshold. Substitution with sugar-sweetened products may adversely affect glycemic control or overall dietary quality.

Interim clinical assessment can document sweetener identity, product or formulation, estimated dose, frequency, and duration together with fiber intake, weight trajectory, metabolic status, and concomitant medications. Oncology dietitian involvement is appropriate for high habitual intake or complex dietary substitution. Counseling can support overall dietary quality and informed preference without delaying or modifying ICI treatment; any NNS-specific advice remains precautionary rather than evidence of proven therapeutic benefit.

### 6.5 Limitations

This structured narrative review remains susceptible to study-selection and publication bias. Heterogeneity in sweetener identity, formulation, carrier ingredients, dose, duration, host characteristics, and analytical methods precluded quantitative synthesis. The evidence framework describes directness and translational distance rather than formal certainty of evidence. Most immune, barrier, and tumor mechanisms derive from animal or model systems under exposure conditions with uncertain correspondence to habitual human intake. The principal NNS–ICI model depends largely on a single sucralose-centered publication and lacks independent prospective clinical validation. Additional gaps include limited power for sex-specific analyses, sparse post-cessation sampling, absence of validated baseline responder signatures, limited direct measurement of candidate metabolites within tumors, uncertainty regarding NNS effects on tumor-resident microbiota, and imperfect transferability of murine and humanized-mouse findings.

## 7 Conclusion

NNS constitute heterogeneous exposures whose biological relevance depends on compound identity, purified versus commercial formulation, carrier matrix, dose, duration, host background, and outcome layer. Human gut microbiota findings remain inconsistent, and clinical ICI evidence comprises an observational sucralose association. Animal and cellular studies provide compound-specific mechanistic evidence with limited clinical directness.

Current evidence does not establish a basis for NNS-specific dietary modification during immune checkpoint inhibitor therapy. The evidence supports compound-specific biological plausibility, an observational sucralose association, and preclinical causality within selected models rather than a clinically proven class-wide effect. Prospective validation requires sweetener-specific and biomarker-supported exposure assessment, vehicle-matched designs, integrated gut microbiota, metabolomic, and immune profiling, prespecified sex and responder analyses, post-cessation sampling, tumor-relevant metabolite measurements, and human–mouse concordance.
