# Supplementary material for: Non-nutritive sweeteners, the gut microbiome, and tumor immunity: evidence hierarchy and a sucralose-centered translational model
Source: Front Nutr. 2026 Aug 18;13:1868431. doi: 10.3389/fnut.2026.1868431 (PMC13531449; doi:10.3389/fnut.2026.1868431)
Supplement: Supplementary file 1 [file Table_1.DOCX]

Table S1. FDA and JECFA acceptable daily intake definitions and standardization rules for non-nutritive sweeteners considered in this review.

| Sweetener | FDA ADI/status | JECFA ADI | Standardization note |
| --- | --- | --- | --- |
| Acesulfame potassium | 15 | 0–15 | Use the agency-specific value; report whether exposure is Ace-K mass. |
| Aspartame | 50 | 0–40 | No parent-compound urinary biomarker; retain agency-specific denominator. |
| Cyclamate | Not permitted as a food additive; no FDA ADI | 0–11 as cyclamic acid | Convert sodium cyclamate ×0.89 or calcium cyclamate ×0.90 to cyclamic-acid equivalents. |
| Saccharin | 15 | 0–5 (group ADI) | State whether intake is reported as saccharin or a salt and preserve the source basis. |
| Sucralose | 5 | 0–15 | FDA- and JECFA-based %ADI values are not interchangeable; state the denominator. |
| Neotame | 0.3 | 0–2 | Use the agency-specific value. |
| Advantame | 32.8 | 0–5 | Use the agency-specific value. |
| Steviol glycosides | FDA consumer table: 12 for rebaudioside A, derived from JECFA value | 0–4 as steviol equivalents | Convert rebaudioside A ×0.33 or stevioside ×0.40 to steviol equivalents. |
| Monk fruit extract | No numerical FDA ADI; selected uses are GRAS | No numerical ADI identified in database search | Do not assign an ADI percentage unless an authoritative numerical denominator is specified. |
| Thaumatin | No numerical FDA ADI; selected uses are GRAS | ADI not specified (group) | Report administered mass and formulation; do not convert “not specified” to a number. |

ADI is expressed as mg/kg body weight/day unless otherwise specified. FDA values and regulatory status were checked against current FDA consumer information ; JECFA values were checked against the WHO JECFA database on 20 July 2026. “Not specified” is a formal JECFA designation and does not mean that unlimited intake is advised.Standardization equations: mg/kg body weight/day = total daily amount (mg) ÷ body weight (kg); %ADI = [exposure (mg/kg body weight/day) ÷ agency-specific ADI] × 100; beverage exposure (mg/kg body weight/day) = concentration (mg/L) × volume consumed (L/day) ÷ body weight (kg). Molecule-specific exposure cannot be back-calculated when commercial formulation composition is unavailable. Values use the chemical basis specified by the regulating agency; FDA- and JECFA-derived %ADI values require a common denominator before pooling.
